# Inferring mechanisms of copy number change from haplotype structures at the human *DEFA1A3* locus

**DOI:** 10.1186/1471-2164-15-614

**Published:** 2014-07-21

**Authors:** Holly A Black, Fayeza F Khan, Jess Tyson, John AL Armour

**Affiliations:** School of Life Sciences, University of Nottingham, Queen’s Medical Centre, Nottingham, NG7 2UH UK

**Keywords:** *DEFA1A3*, CNV, Defensin, Structural haplotype

## Abstract

**Background:**

The determination of structural haplotypes at copy number variable regions can indicate the mechanisms responsible for changes in copy number, as well as explain the relationship between gene copy number and expression. However, obtaining spatial information at regions displaying extensive copy number variation, such as the *DEFA1A3* locus, is complex, because of the difficulty in the phasing and assembly of these regions. The *DEFA1A3* locus is intriguing in that it falls within a region of high linkage disequilibrium, despite its high variability in copy number (n = 3–16); hence, the mechanisms responsible for changes in copy number at this locus are unclear.

**Results:**

In this study, a region flanking the *DEFA1A3* locus was sequenced across 120 independent haplotypes with European ancestry, identifying five common classes of *DEFA1A3* haplotype. Assigning *DEFA1A3* class to haplotypes within the 1000 Genomes project highlights a significant difference in *DEFA1A3* class frequencies between populations with different ancestry. The features of each *DEFA1A3* class, for example, the associated *DEFA1A3* copy numbers, were initially assessed in a European cohort (n = 599) and replicated in the 1000 Genomes samples, showing within-class similarity, but between-class and between-population differences in the features of the *DEFA1A3* locus. Emulsion haplotype fusion-PCR was used to generate 61 structural haplotypes at the *DEFA1A3* locus, showing a high within-class similarity in structure.

**Conclusions:**

Structural haplotypes across the *DEFA1A3* locus indicate that intra-allelic rearrangement is the predominant mechanism responsible for changes in *DEFA1A3* copy number, explaining the conservation of linkage disequilibrium across the locus. The identification of common structural haplotypes at the *DEFA1A3* locus could aid studies into how *DEFA1A3* copy number influences expression, which is currently unclear.

**Electronic supplementary material:**

The online version of this article (doi:10.1186/1471-2164-15-614) contains supplementary material, which is available to authorized users.

## Background

Copy number variation (CNV), involving a deletion or duplication of a region of DNA ≥1 kb in length, is a common feature of the human genome [1–4]. At some loci, recurrent deletion and duplication events lead to a region being present in a highly variable number of copies; these are referred to as multiallelic copy number variants. Multiallelic CNV has been associated with a variety of disease phenotypes [5–11]. However, an increase in copy number does not always result in increased mRNA levels and, in turn, an increased protein production [12]. In these circumstances, the knowledge of the structure of the locus can be essential for understanding the effect of CNV on phenotype. For example, in the case of the association of low *FCGR3B* copy number with systemic lupus erythematosus (SLE), it is the presence of a zero-copy *FCGR3B* haplotype, which leads to aberrant expression of a chimeric gene, *FCGR2B’*, in natural killer cells, that may be the key factor in an increased SLE risk, and not a reduced dosage of *FCGR3B* [13, 14]. Therefore, understanding the effect of copy number variation on phenotype comes from not only knowing the copy number of a region, but the spatial arrangement of the locus.

**Figure 1:**
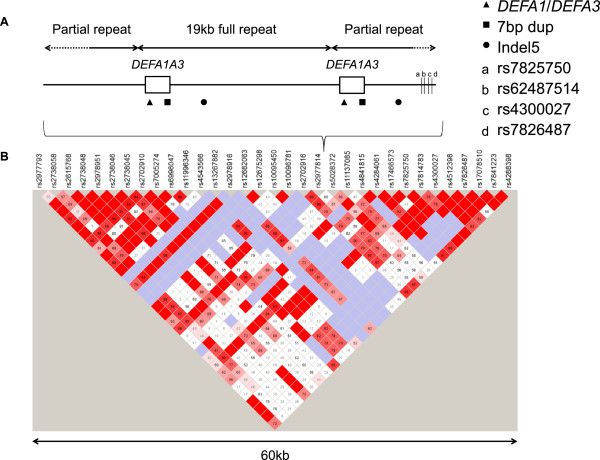
**Structure of the** ***DEFA1A3*** **locus. A)** The *DEFA1A3* locus consists of two single-copy partial repeats surrounding a variable number of full repeats. Each of the full repeats and the centromeric partial repeat contain a gene locus occupied by either *DEFA1* or *DEFA3*. Symbols show the positions of the variant distinguishing *DEFA1* from *DEFA3*, a 7 bp duplication in intron 1 of each copy of *DEFA1A3* and a 5 bp Indel located upstream of each copy of *DEFA1A3*. The positions of the four SNPs tagging *DEFA1A3* haplotype class are shown. Adapted from Khan *et al.*[18]. **B)** There are SNPs either side of the *DEFA1A3* locus displaying high levels of linkage disequilibrium (D’ = 1), as shown by phased SNP genotype data for the HapMap CEU1 individuals, downloaded from the HapMap project (release #24, phase 1 and 2) [35, 54]. D’ values are shown.

One locus exhibiting multiallelic CNV is the α-defensin *DEFA1A3* locus on human chromosome 8p23.1 (Figure 1A) [15–18], with individuals having between 3–16 copies of *DEFA1A3* [17–20]. SNPs are usually poor tags of copy number at multiallelic loci, due to the limited ability of a biallelic SNP to tag multiple different copy number states [21]. However, the SNP rs4300027 has been identified as a tag of *DEFA1A3* copy number in populations with European ancestry, an association which has not been shown in other populations [18]. At the locus, each *DEFA1A3* repeat unit can be occupied by one of two α-defensin genes, either *DEFA1* or *DEFA3*, adding additional complexity. The two genes encode the human neutrophil peptides (HNP) 1–3; these are antimicrobial peptides involved in the innate immune response [22–25]. A recent GWAS found the SNP rs2738048, which falls within the same linkage disequilibrium block as *DEFA1A3*, to be associated with risk of IgA nephropathy in the Han Chinese population [26]. The basis of this association is unknown, but highlights a need to understand how variation at the *DEFA1A3* locus influences HNP1-3 expression. There has only been a single small-scale study comparing *DEFA1A3* copy number with HNP1-3 expression, which identified a positive correlation [19]. However, the spatial arrangement of the locus may influence expression.

Non-allelic homologous recombination (NAHR) is the predominant mechanism through which multiallelic copy number variants are formed, requiring segmental duplications (SDs, also referred to as low copy repeats, LCRs) of ≥10 kb in length with ≥95% sequence identity to mediate the rearrangements [4, 27, 28]. NAHR is a type of homologous recombination occurring between non-allelic copies of a region in different chromosomal positions, resulting in deletions and duplications and can lead to either gene conversion or chromosomal crossover [29–32]. However, there are SNPs either side of the *DEFA1A3* locus that display high levels of linkage disequilibrium (LD) (Figure 1B). This suggests that crossover events across the *DEFA1A3* region are rare, despite its high variability in copy number. In addition, it is unclear what features of the *DEFA1A3* locus are shared between related haplotypes- for example, are haplotypes with the same *DEFA1A3* copy number more closely related than haplotypes with different *DEFA1A3* copy numbers? Therefore, the mechanisms responsible for generating variation at the *DEFA1A3* locus are unclear.

In this study, flanking sequence variation was used to identify related haplotypes at the *DEFA1A3* locus, in order to determine the shared features of these haplotypes, such as *DEFA1A3* copy number. Previously, we have demonstrated that emulsion haplotype fusion PCR (EHF-PCR) can be used to determine the relative positions of the *DEFA1* and *DEFA3* genes across a haplotype, providing spatial information at the *DEFA1A3* locus [33]. This technique has now been applied to a larger number of haplotypes and to additional variants within the *DEFA1A3* locus to generate more detailed structural haplotypes, allowing an inference of the mechanisms responsible for changes in *DEFA1A3* copy number. This has provided a comprehensive understanding of the common allelic structures of the *DEFA1A3* locus present in populations with European ancestry.

## Results

### Gene conversion events at *DEFA1A3*

**Figure 2:**
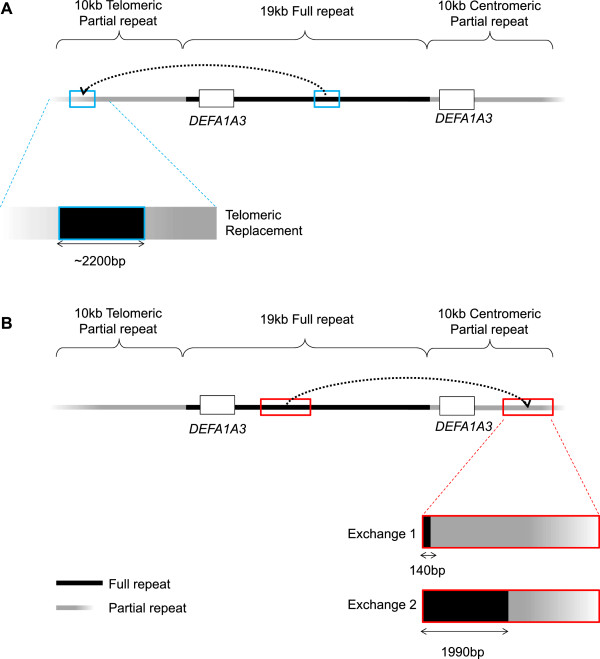
**Gene conversion events at** ***DEFA1A3.*** **A)** The *DEFA1A3* full and partial repeats are highly similar in their sequence, such that the partial repeats can be aligned with the full repeats (blue boxes). However, there are sequence differences between the *DEFA1A3* full and partial repeats. Array CGH data and PCR analysis identified a sequence replacement in the *DEFA1A3* telomeric partial repeat, in which the sequence from the equivalent region of the *DEFA1A3* full repeats replaced the sequence in the telomeric partial repeat location. This occurred over approximately a 2.2 kb interval and is referred to as the “telomeric replacement polymorphism”. **B)** Sequence data for a 4.1 kb region within the *DEFA1A3* centromeric partial repeat (red box) identified two additional gene conversion events- a ~140 bp event termed “Exchange 1” and a ~1990 bp event termed “Exchange 2”.

Our analysis of array-CGH data from Conrad *et al*. [34] appeared to identify a deletion polymorphism in the telomeric partial repeat of the *DEFA1A3* locus. Further investigation in this study has demonstrated that this is not a deletion, but a replacement of the *DEFA1A3* telomeric partial repeat sequence with sequence from the equivalent region of the *DEFA1A3* full repeats, in a gene conversion event. This is the “Telomeric Replacement Polymorphism” (Figure 2A). The telomeric boundary of the replacement is within the interval GRCh37/hg19 chr8: 6825864–6825878 and the centromeric boundary within the interval chr8: 6828055–6828082; therefore, it covers a region of approximately 2.2 kb. 16 of the 120 HapMap CEU1 haplotypes (13%) carry the telomeric replacement polymorphism.

Sequence data obtained from the centromeric partial repeat (GRCh37/hg19 chr8: 6876778–6880877) identified two similar events (Figure 2B). In each case, the sequence in the centromeric partial repeat location has been replaced by sequence from the equivalent region of the full repeats. Exchange 1 haplotypes contain a replacement extending over at least 140 bp. The centromeric boundary of the replacement falls within the region chr8: 6876928–6876969. The telomeric boundary extends beyond chr8: 6876788, although it may be continuous with the block of full repeats. Exchange 2 haplotypes contain a replacement extending over at least 1990 bp. The centromeric boundary of the replacement falls within the region chr8: 6878778–6879044. The telomeric boundary extends beyond chr8: 6876788, although it may be continuous with the block of full repeats.

### *DEFA1A3*haplotype classes

Sequence similarity across the 4.1 kb centromeric flanking region allowed the identification of five different classes of *DEFA1A3* haplotype, in which haplotypes within each class shared identical or highly similar flanking sequence, which was distinct from the sequence of haplotypes within the other classes. These five classes are: Reference Sequence (the sequence found in the GRCh37/hg19 human reference assembly), Class 1, Class 2 (each contains multiple unique sequence differences compared to the Reference Sequence), Exchange 1 (contains the Exchange 1 sequence replacement polymorphism) and Exchange 2 (contains the Exchange 2 sequence replacement polymorphism).

**Figure 3:**
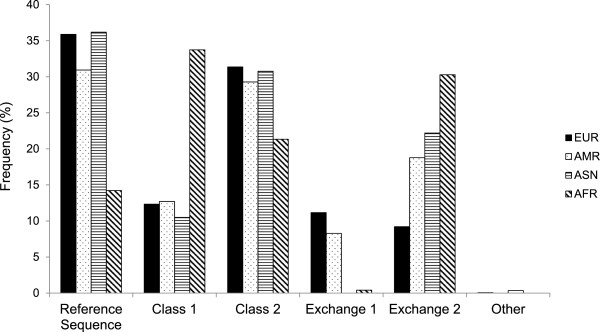
***DEFA1A3*** **haplotype class frequencies.** The frequency distribution of the *DEFA1A3* haplotype classes across different worldwide populations, based on the 3218 independent haplotypes observed across the HapMap CEU, ECACC HRC and 1000 Genomes samples.

Four SNPs were identified, through a combination of sequencing and analysis of phased HapMap data [35] (see Methods), which, due to their pattern of LD, are able to tag the five *DEFA1A3* haplotype classes (Figure 1, Table 1 and Additional file 1: Table S1). The diploid *DEFA1A3* class genotype was identified for an additional 539 independent European individuals (HapMap CEU2 and ECACC HRC1-5) and the *DEFA1A3* haplotype class was identified for the 2184 haplotypes from the 1000 Genomes project. Across the 3216 independent haplotypes sampled, only 3 exceptions to the expected pattern of LD were observed; one ECACC HRC haplotype and two 1000 Genomes project Asian (ASN) haplotypes. The frequency distribution of the haplotype classes varies significantly worldwide (Figure 3); χ^2^ = 362, p = 6.08 × 10^-68^. In the1000 Genome ASN, American (AMR) and European (EUR) samples, the Reference Sequence class is the most frequent, with Class 2 the next most common class. However, this is not the case in the 1000 Genomes African (AFR) samples, where Class 1 is by far the most common *DEFA1A3* haplotype class. The EUR samples have a low frequency of Exchange 2, compared to the worldwide frequency of Exchange 2, whilst Exchange 1 is absent, or at a very low frequency, in the AFR and ASN samples.

**Table 1:** **Linkage disequilibrium of** ***DEFA1A3*** **flanking SNPs**

Haplotype class	SNP Genotypes			
	rs4300027	rs7825750	rs7826487	rs62487514
Reference Sequence	T	T	A	C
Class 1	C	T	G	C
Class 2	C	C	A	C
Exchange 1	T	T	A	A
Exchange 2	C	T	A	C

The genotype combinations for the four tag SNPs able to identify each of the five *DEFA1A3* haplotype classes. Due to the pattern of linkage disequilibrium (Additional file 1: Table S1), a diploid genotype profile can be used to determine the haplotype classes of the two haplotypes for the genotyped individual.

### Features of *DEFA1A3*haplotype classes

The SNP rs4300027 has previously been identified as a tag of *DEFA1A3* copy number in the European population (p = 1.3×10^-45^), with the C allele tagging haplotypes with a low *DEFA1A3* copy number (2–3 copies) and the T allele tagging haplotypes with a high *DEFA1A3* copy number (4–5 copies) [18]. We investigated whether a combination of four SNP genotypes may further partition this association or identify tags of additional features. Therefore, the diploid *DEFA1A3* haplotype class (e.g. Class 1 homozygous/ heterozygous/ negative) was compared to features of the *DEFA1A3* copy number variable region using either a Chi Square or Cochran Armitage test, for 599 unrelated European individuals (Table 2 and Additional file 1: Table S2). The diploid *DEFA1A3* copy number, as well as the ratio of the number of copies of *DEFA1* versus *DEFA3*, inserted to deleted form of a 5 bp Indel (Indel5) and unduplicated to duplicated form of a 7 bp duplication (7 bp dup) are known for these samples (Figure 1A) [18], allowing a comparison between *DEFA1A3* haplotype class and multiple variants at the locus.

**Table 2:** **Comparing** ***DEFA1A3*** **haplotype class with features of the locus in individuals with European ancestry**

Haplotype class	***DEFA1A3***copy number	***DEFA3***frequency	Indel5 insertion frequency	7 bp duplication frequency
Reference Sequence	6×10^-26^; high	1×10^-7^; high	2×10^-5^; low	3×10^-11^; low
Class 1	2×10^-11^; low	4×10^-9^; low	3×10^-5^; high	NS
Class 2	1×10^-21^; low	1×10^-12^; high	2×10^-10^; low	NS
Exchange 1	3×10^-3^; high	2×10^-14^; low	1×10^-39^; high	2×10^-39^; high
Exchange 2	NS	3×10^-5^; low	2×10^-9^; low	NS

p-values and direction of significant associations obtained for Chi Square or Cochran-Armitage tests (Additional file 1: Table S2) comparing *DEFA1A3* haplotype class with features of the *DEFA1A3* locus, based on the HapMap CEU and ECACC HRC samples. All p-values were adjusted for multiple testing using Bonferroni correction. NS = not significant. High = associated with a high *DEFA1A3* copy number/allele frequency; Low = associated with a low *DEFA1A3* copy number/allele frequency.

This demonstrates that each *DEFA1A3* haplotype class has its own unique profile of features. Both the Reference Sequence and Exchange 1 haplotypes are associated with a high *DEFA1A3* copy number, whilst Class 1 and Class 2 are associated with a low *DEFA1A3* copy number (Table 2). This forms the basis of the previously reported association between the SNP rs4300027 and *DEFA1A3* copy number in the European population, with Reference Sequence and Exchange 1 haplotypes having the T allele and Class 1 and Class 2 haplotypes the C allele. However, haplotype-specific copy number information, obtained by Khan *et al.* demonstrates that each *DEFA1A3* haplotype class is not associated with a single copy number state [18]. For example, whilst Exchange 1 haplotypes are generally associated with a high copy number, Exchange 1 haplotypes with between 2–7 copies have been observed in the HapMap CEU1 population (data not shown) [18]. In addition to associations with *DEFA1A3* copy number, all five classes show an association with the frequency of the *DEFA3* gene and the Indel5 insertion, whilst only two *DEFA1A3* haplotype classes are significantly associated with the frequency of the 7 bp duplication (Table 2).

However, these associations are based solely on the European population and it is not clear if the same features can be extended to other worldwide populations. Whilst information for the three internal allelic variants (*DEFA1*/*DEFA3*, Indel5 and 7 bp duplication) was unavailable, the *DEFA1A3* copy number has been estimated for 1047 of the 1092 individuals within the 1000 Genomes project. This allowed a comparison of *DEFA1A3* haplotype class with *DEFA1A3* diploid copy number in non-European populations (Table 3 and Additional file 1: Table S3). The 1000 Genome AMR samples show an association of Class 2 with a low *DEFA1A3* copy number and Reference Sequence and Exchange 1 with a high copy number, the same as was observed in the CEU and HRC samples. However, the association of Class 1 and a low copy number, which was observed for the European samples, is not replicated in the AMR samples. Despite this, the SNP rs4300027 is still significantly associated with *DEFA1A3* copy number in the AMR samples (p = 7×10^-10^). The ASN samples also show associations of Class 2 with a low *DEFA1A3* copy number and of Reference Sequence with a high copy number, as well as of Exchange 2 with a low copy number. Whilst this mirrors the association with rs4300027 observed in the EUR samples, Class 1 is significantly associated with a high *DEFA1A3* copy number in the ASN samples, leading to a weaker association between rs4300027 genotype and *DEFA1A3* copy number (p = 5×10^-4^). There are no significant associations between haplotype class and *DEFA1A3* copy number in the AFR samples, suggesting high within-class variability in copy number and explaining the observation that the SNP rs4300027 does not tag copy number in this population (p = 0.114). The 1000 Genome dataset also provides information on additional EUR samples. The associations identified differ from those observed previously; although the association between Class 2 and a low *DEFA1A3* copy number and Reference Sequence with a high copy number are observed, Class 1 and Exchange 1 show no association with *DEFA1A3* copy number and a novel association of Exchange 2 with a low copy number is observed.

**Table 3:** **Comparing** ***DEFA1A3*** **haplotype class with copy number in 1000 Genome individuals**

Haplotype class	Africa	America	Asia	Europe
Reference Sequence	NS	3×10^-4^; high	0.009; high	2×10^-26^; high
Class 1	NS	NS	9×10^-16^; high	NS
Class 2	NS	1×10^-6^; low	1×10^-8^; low	1×10^-18^; low
Exchange 1	-	0.003; high	-	NS
Exchange 2	NS	NS	0.045; low	3×10^-4^; low

p-values and direction of significant associations obtained for Chi Square or Cochran-Armitage tests (Additional file 1: Table S3) comparing *DEFA1A3* haplotype class with *DEFA1A3* copy number, based on the 1000 Genomes samples. All p-values were adjusted for multiple testing using Bonferroni correction. NS = not significant. - = no test performed (see Additional file 1: Table S3). High = associated with a high *DEFA1A3* copy number; Low = associated with a low *DEFA1A3* copy number.

### *DEFA1A3*haplotype structures

**Figure 4:**
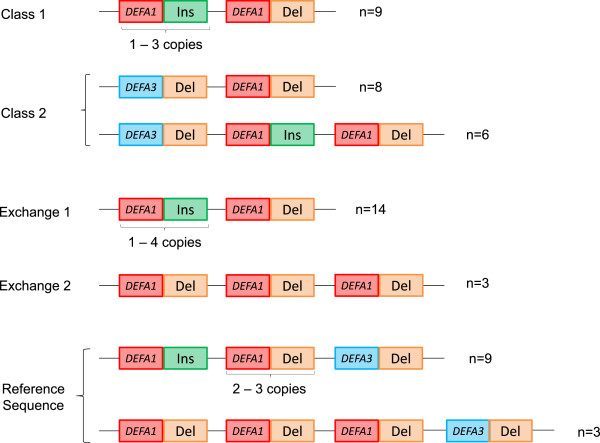
***DEFA1A3*** **haplotype structures.** The common *DEFA1A3* haplotype structures observed in haplotypes with European ancestry (HapMap CEU1) obtained using EHF-PCR and sequencing. “Del” and “Ins” refer to the deleted and inserted forms of the Indel5 variant. 52 of the 61 haplotypes observed (85%) conformed to one of these structures.

Emulsion haplotype fusion PCR (EHF-PCR) has previously been applied at the *DEFA1A3* locus to determine the relative positions of the *DEFA1* and *DEFA3* genes across a haplotype [33]. By applying this technique to additional variants, a more detailed picture of the haplotype structures of the locus can be determined, allowing the mechanisms of change in copy number at *DEFA1A3* to be identified. For 84 independent haplotypes in the HapMap CEU1 population, the *DEFA1A3* copy number, as well as the ratio of *DEFA1 vs. DEFA3* and inserted to deleted form of Indel5 are known [18]. The positions of these allelic variants were determined for 61 of these 84 haplotypes using EHF-PCR and sequencing (Figure 4). The centromeric-most Indel5 site is located within the sequenced flanking region (GRCh37/hg19 chr8: 6876778–6880877) and as such, the centromeric-most position was captured for all 120 haplotypes. In this location, only the deleted form was observed.

The structures of 17 Reference Sequence haplotypes were determined, 12 of which display one of the structures shown in Figure 4. Whilst there are many different structural haplotypes, with 4 or 5 copies of *DEFA1A3*, the structures are highly similar to each other, with single copy differences between most structures that could be accounted for by a single deletion or duplication event. Two of the haplotypes with structures that do not fit this pattern have only two copies of *DEFA1A3*, which is unusual for haplotypes within the Reference Sequence class, whilst the three others have the Indel5 insertion at the second repeat from the telomeric end, suggesting a structural rearrangement.

For Class 1, 9 of the 11 haplotypes observed display a structure shown in Figure 4, in which there are a variable number of copies of a repeat unit containing *DEFA1* and the Indel5 insertion. In this case, not only do the haplotypes share a similar structure, but the repeat units are also highly similar. The two haplotypes which do not fit this pattern both have a copy of the *DEFA3* gene, which is usually absent from Class 1 haplotypes.

For Class 2, 14 of the 15 haplotypes observed display a structure shown in Figure 4. Again, there is a single deletion or duplication event that could account for the differences between the haplotypes. The haplotype which does not fit this pattern lacks *DEFA3*, which is uncommon for Class 2 haplotypes.

For Exchange 1, 14 of the 15 haplotypes display the structure shown in Figure 4; these are similar to Class 1 haplotypes, but vary in copy number from 1–5 copies. Again, it is not just the structures, but the repeat units that are highly similar. The haplotype that does not fit this pattern includes the *DEFA3* gene, which is usually absent from Exchange 1 haplotypes. There are only three examples of Exchange 2 haplotypes analysed, all with the same structure and with all three repeats containing both *DEFA1* and the Indel5 deletion.

Although there is within-class variation, the structures identify common features of each *DEFA1A3* class. For example, if Reference Sequence haplotypes contain a copy of the *DEFA3* gene, it is in the centromeric-most copy, whereas for Class 2 haplotypes, it is in the telomeric-most copy. For the Indel5 variant, both Class 1 and Exchange 1 haplotype have the deletion allele in the centromeric-most copy of the array. For Reference Sequence haplotypes, the Indel5 insertion allele is always in the telomeric-most copy, whereas for Class 2, it is in the middle copy of three-copy haplotypes.

## Discussion

In order to fully understand the relationship between multiallelic CNV and expression, it is necessary to not only reliably genotype the copy number, but to understand the positions of these copies across a haplotype. This is especially true at a locus like *DEFA1A3*, in which each repeat unit in the array can be occupied by one of two different genes. Through the use of flanking sequence information, five common *DEFA1A3* haplotype classes have been identified, each of which has specific associations with internal variants in populations of European ancestry. A small-scale analysis on 1000 Genomes samples demonstrates between-population differences within each *DEFA1A3* haplotype class. This is due to the combination of the different copy number distributions associated with each haplotype class and the varying frequencies of the classes between populations.

The use of EHF-PCR to provide spatial information at the *DEFA1A3* locus has been expanded in this study, to look not only at the positions of the *DEFA1* and *DEFA3* genes across a haplotype, but also the positions of an additional allelic variant, Indel5. Although the Indel5 variant does not necessarily change the expression or function of HNP1-3, it provides an additional landmark across a haplotype, allowing a more detailed comparison of haplotype structures. In total, structural haplotypes were obtained for 61 independent haplotypes within the HapMap CEU1 population. The CEU1 population sample appears to be representative of the wider European cohort in terms of the associations between *DEFA1A3* class and features of the locus (data not shown). Therefore, the structures observed should be representative of haplotypes with European ancestry. The structures observed show that haplotypes within each *DEFA1A3* class have highly similar structures, despite having different copy numbers. In addition, some classes have multiple copies of a repeat unit containing the same gene and Indel5 allele, which was expected, given that all five *DEFA1A3* classes are significantly associated with either a high or low frequency of both *DEFA3* and the Indel5 insertion. This repeat unit similarity is likely to promote NAHR, which relies on high sequence identity to facilitate rearrangements. Given that the *DEFA1A3* locus falls within a region of high LD, this information suggests that the major mechanisms for copy number change at the *DEFA1A3* locus involve intra-allelic rearrangements- i.e. NAHR between haplotypes from the same *DEFA1A3* haplotype class. This process would allow changes in *DEFA1A3* copy number, via NAHR resulting in chromosomal crossover, but would preserve the surrounding LD, as rearrangements would occur between haplotypes within the same *DEFA1A3* class. This presumably results from a bias towards NAHR between sister chromatids, rather than between homologous chromosomes, during meiosis. A bias for NAHR between sister chromatids has been observed previously at the tandemly duplicated human alpha satellite DNA [36], as well as a bias towards NAHR between homologue chromosomes at the a1 locus in maize [37].

A clear example of intra-allelic NAHR is observed in the Exchange 1 class, which is expected to be younger than the four other classes, given that it is absent from the Asian population and is very rare in the African population. Exchange 1 haplotypes with between 2 and 7 copies have been observed and the structures identified in samples with European ancestry show there are variable numbers of copies of a repeat unit with the same gene and Indel5 allele.

However, intra-allelic rearrangements will not be the only mechanism operating at the locus. Inter-allelic rearrangements will occur, but given the conservation of LD across the *DEFA1A3* locus, it is likely that inter-allelic NAHR more often results in gene conversion than chromosomal crossover. This study has identified three gene conversion events occurring in the flanking regions of the *DEFA1A3* locus, supporting this idea. In addition, the vast majority of Class 1 haplotypes lack *DEFA3*, but *DEFA3*-positve Class 1 haplotypes have been observed and this is likely to have resulted from an introduction of *DEFA3* to a Class 1 background, via gene conversion. This idea is consistent with previous studies identifying gene conversion events at variable number tandem repeat loci [38–42], to which *DEFA1A3* is comparable, given it contains multiple copies of a repeat unit with high sequence similarity, positioned in tandem. Gene conversion will homogenise repeat units, which in turn will facilitate further rearrangement events.

Many studies at regions of CNV fail to identify a robust association between copy number and disease risk. This is due to a combination of two factors. Firstly, many studies fail to accurately measure multiallelic copy number, leading to an association that cannot be reproduced [43–47]. Secondly, there is an expectation of a linear relationship in which an increase in copy number results in a proportionate increase in protein expression; however, this is not always the case [12]. As shown for the *FCGR3B* and *NBPF23* loci, knowledge of the allelic structures of the region may be necessary to determine how CNV influences gene expression [13, 14, 48]. Despite accurate measurement of *DEFA1A3* copy number [18], the relationship between gene copy number and expression at this locus remains unclear. Although a positive correlation between *DEFA1A3* copy number and HNP1-3 expression has been reported previously [19], this was a small-scale study. Structural information may be required to fully understand the relationship between *DEFA1A3* copy number and HNP1-3 expression, as well as explain the association between the *DEFA1A3* locus and IgA nephropathy risk [26]. In samples with European ancestry, haplotypes within each *DEFA1A3* class have highly similar structures. Therefore, the simple genotyping of the four SNPs which tag *DEFA1A3* haplotype class, identified in this study, will be sufficient for inferring haplotype structures for haplotypes with European ancestry. This approach could easily be applied to studies comparing *DEFA1A3* structure with HNP1-3 expression or association with a disease phenotype. Therefore, the use of structural information, as derived here for *DEFA1A3*, should be applied to other copy number variable loci, in order to explain associations between the variation observed and protein expression. This may, in turn, aid the understanding of the features of a copy number variable locus that influence disease risk.

## Conclusions

We have defined five common classes of haplotype at the *DEFA1A3* locus. Each class is associated with particular features of the *DEFA1A3* locus and these associations differ between populations. Structural haplotypes have been obtained across the *DEFA1A3* locus for 61 haplotypes with European ancestry, allowing the identification of the common allelic structures at *DEFA1A3*. The structures suggest that intra-allelic rearrangement is the predominant mechanism resulting in copy number variation at the *DEFA1A3* locus.

## Methods

### DNA samples

180 HapMap phase I (CEU1) and II (CEU2) samples [49] and 480 Human Random Control (HRC; panels 1–5) unrelated UK samples from the European Collection of Cell Cultures (ECACC) [50] were used for the study. The DNA was extracted from lymphoblastoid cell lines.

### Measuring *DEFA1A3*copy number

Diploid *DEFA1A3* copy number was measured for the 180 HapMap and 480 ECACC HRC samples and haplotype *DEFA1A3* copy numbers were defined for 84 haplotypes from the HapMap CEU1 population as described by Khan *et al.* [18]. The diploid *DEFA1A3* copy number was estimated for 1047 samples within the 1000 Genomes project using read depth analysis of whole genome sequence data [51]. Raw read data was downloaded from the 1000 Genomes project [51, 52]. The reads mapping to the *DEFA1A3* locus (GRCh37/hg19 chr8: 6829298–6837591, 6848458–6856701 and 6867561–6875800) and two single-copy flanking regions (GRCh37/hg19 chr8: 6700000–6830000 and 6900000–7000000) were counted using Samtools [53], with the command samtools view –c. Flanking regions were selected to have a similar GC content to the copy number variable region selected from the *DEFA1A3* locus. The ratio of reads per base for the *DEFA1A3* locus to the reads per base for the flanking regions was obtained and multiplied by two to give the diploid *DEFA1A3* copy number estimation, which was rounded to the nearest integer value. The 1047 samples consist of individuals with European (EUR) (n = 364), Asian (ASN) (n = 280), African (AFR) (n = 228) and American (AMR) (n = 175) ancestry. EUR = CEU + FIN + GBR + IBS + TSI. ASN = CHB + CHS + JPT. AFR = ASW + LWK + YRI. AMR = CLM + MXL + PUR. A comparison with copy numbers estimated by Khan *et al.* [18] shows read depth provides an accurate estimation of *DEFA1A3* copy number (supplementary methods and Additional file 1: Figure S1).

### Identification of *DEFA1A3*haplotype classes

A 4.1 kb region immediately centromeric to the *DEFA1A3* locus (GRCh37/hg19 chr8: 6876778–6880877) was resequenced across the 30 HapMap CEU1 trios, allowing complete phased haplotype sequences to be obtained using segregation or allele-specific PCR (supplementary methods and Additional file 1: Table S4). The primers designed to amplify the region were designed to ensure amplification specifically from the *DEFA1A3* centromeric partial repeat. PCR products were purified using AmpureXP (Agencourt), according to the manufacturer’s protocol. Approximately 20 ng of purified PCR product was Sanger sequenced using Big Dye (Invitrogen), according to the manufacturer’s protocol. Sequenced products were cleaned using CleanSeq (Agencourt) according to the manufacturer’s protocol and analysed using an ABI 3730. Twelve primers were used to sequence the region (Additional file 1: Table S5).

### Genotyping of *DEFA1A3*haplotype classes

Flanking sequence information allowed the identification of five major *DEFA1A3* haplotype classes. A combination of four SNPs is able to tag these five *DEFA1A3* haplotype classes; three SNPs were identified from sequencing, as described above, whilst the fourth, rs7826487, was identified from phased HapMap genotype data [35, 54]. A PCR-RFLP assay was used to genotype each SNP across the HapMap CEU2 and ECACC HRC samples. The SNP rs4300027 was genotyped as described by Khan *et al.* [18]. The primers and cycling conditions for the other three assays are shown in Additional file 1: Table S6. All assays used 1 μM each primer, 0.5 Units *Taq* DNA polymerase (NEB), 10 ng genomic DNA and a standard buffer, with final reaction concentrations of 50 mM Tris HCl pH8.8, 12.5 mM Ammonium Sulphate, 1.4 mM MgCl_2_, 7.5 mM 2-mercaptoethanol, 200 μM each dNTP and 125 μg/ml BSA. The same four SNPs were genotyped as part of the 1000 Genomes project*,* allowing *DEFA1A3* haplotype class to be assigned to the 2184 haplotypes within the 1000 Genomes dataset [51, 55].

### Telomeric replacement polymorphism

A three-primer assay was designed to genotype the telomeric replacement polymorphism in the HapMap CEU1 individuals. The forward primer AGCAGCAGATCCGGTATAATC produces a 645 bp product with the reverse primer AGAGCCCAATAAATCTAACAGG from non-replacement haplotypes or a 453 bp product with the reverse primer GACTCGTTCTTTCTGGATTCAC from haplotypes carrying the replacement. The cycling conditions consisted of an initial denaturation at 95°C for 3 minutes, followed by 36 cycles of 95°C for 30 seconds, 60°C for 30 seconds and 70°C for 30 seconds. Each 10 μl reaction contained 1 μM each primer, 0.5 Units *Taq* DNA polymerase (NEB), 10 ng genomic DNA and a standard buffer, as described above.

### Statistical analysis

A series of Chi Square and Cochran-Armitage tests were used to compare *DEFA1A3* haplotype class with features of the *DEFA1A3* locus. The copy number and frequency categories were designated such that each category was comparably populated. For Class 1 and Exchange 2, homozygous and heterozygous individuals were grouped, due to their low frequencies. Individuals were counted multiple times; for example, an individual homozygous positive for the Reference Sequence would have also been counted as homozygous negative for Class 1, Class 2, Exchange 1 and Exchange 2. To account for this, p-values were adjusted using Bonferroni correction.

### Emulsion haplotype fusion PCR

Four emulsion haplotype fusion PCR (EHF-PCR) systems were used, based on a design described by Tyson and Armour [33]. Two single-copy flanking regions, one centromeric and one telomeric to the *DEFA1A3* locus, were fused to the region containing the variant distinguishing *DEFA1* from *DEFA3* (termed “telomeric gene” and “centromeric gene” respectively) or the region containing the Indel5 variant (termed “telomeric Indel5” and “centromeric Indel5”). The telomeric flanking region contains four SNPs (rs2978951, rs2738046, rs2738045 and rs2702910), the phased genotype data for which was obtained from the HapMap project [35, 54]. The centromeric flanking region contains three SNPs (rs4300027, rs4512398 and rs17382102), the phased genotype data for which was obtained from sequencing in this work.

The 25 μl aqueous phase for each EHF-PCR contained 1× Phusion GC buffer (NEB), 0.2 mM each dNTP, 1 μM F1 primer, 25nM F2’R1 primer, 1 μM R2 primer, 2 Units Phusion DNA polymerase (NEB) and 50 ng genomic DNA. The preparation of the emulsion was adapted from a method first described by Turner and Hurles [56]. The aqueous phase was aliquoted to 0.5 ml PCR tubes, to which 50 μl silicone oil (described in [56]) was added. A 3 mm tungsten carbide bead (Qiagen) was added to the lid of the tube and the tube was closed such that it remained in an inverted position; otherwise, the bead remains in a constrained space at the bottom of the tube during vortexing. Inversion of the tube is essential for emulsion formation. The inverted tube was vortexed at speed 5 for 1 minute 30 seconds using a Vortex Genie 2, to give aqueous droplets approximately 5 μM in size (data not shown). The primers and cycling conditions are shown in Additional file 1: Table S7.

25 μl of 1× Phusion GC buffer (NEB) was added to each sample post-PCR to increase the volume of the aqueous phase; this was recovered as described by Tyson and Armour [33]. Allele-specific reamplification of the fusion products was performed to allow haplotype-specific sequencing of the fused region. The primers and cycling conditions used are shown in Additional file 1: Table S8. All reamplifications were performed in a 20 μl reaction containing 1× NH_4_ buffer (Bioline), 2 mM MgCl_2_, 0.2 mM each dNTP, 0.5 μM each primer, 1 Unit *Taq* DNA polymerase (Bioline) and 1 μl PCR template. The exception was reamplifications using centromeric Indel5 products, for which the 20 μl reaction contained 0.5 μM each primer, 1 Unit *Taq* DNA polymerase, 1 μl PCR template and 1× standard buffer, as described above. The reamplified products were purified and sequenced, as described above.

## Availability of supporting data

The data sets supporting the results of this article are included in Additional files 2, 3, 4, 5 and 6.

## Electronic supplementary material

Additional file 1: **Supplementary methods, figures and tables.** Contains additional information explaining the methods used to phase the *DEFA1A3* centromeric flanking region and how read depth was used to estimate *DEFA1A3* copy number, as well as supplementary Tables 1–8 and supplementary Figure 1. (PDF 174 KB)

Additional file 2: **Data file.** Includes the data used for analysis in this publication: *DEFA1A3* copy numbers estimated for 1047 of the 1000 Genomes samples using read depth; SNP genotypes and the *DEFA1A3* haplotype classes assigned to the HapMap CEU and ECACC HRC samples (rs4300027 genotypes from Khan *et al.* [18]); Telomeric Replacement Polymorphism genotypes for the HapMap CEU1 samples; haplotype structures for 61 HapMap CEU1 haplotypes. (XLSX 85 KB)

Additional file 3: ***DEFA1A3*** **read depth copy number estimates.** *DEFA1A3* copy numbers estimated for 1047 of the 1000 Genomes samples using read depth. (CSV 20 KB)

Additional file 4: ***DEFA1A3*** **haplotype classes.** SNP genotypes and the *DEFA1A3* haplotype classes assigned to the HapMap CEU and ECACC HRC samples (rs4300027 genotypes from Khan *et al.* [18]). (CSV 23 KB)

Additional file 5: **Telomeric replacement polymorphism.** Telomeric Replacement Polymorphism genotypes for the HapMap CEU1 samples. (CSV 879 bytes)

Additional file 6: ***DEFA1A3*** **haplotype structures.** Haplotype structures for 61 HapMap CEU1 haplotypes. (CSV 5 KB)
