# Supplementary material for: Inferring mechanisms of copy number change from haplotype structures at the human DEFA1A3 locus
Source: BMC Genomics. 2014 Jul 21;15(1):614. doi: 10.1186/1471-2164-15-614 (PMC4117965; doi:10.1186/1471-2164-15-614)
Supplement: Supplementary file 1 — Additional file 1: Supplementary methods, figures and tables. Contains additional information explaining the methods used to phase the DEFA1A3 centromeric flanking region and how read depth was used to estimate DEFA1A3 copy number, as well as supplementary Tables 1–8 and supplementary Figure 1. (PDF 174 KB) [file 12864_2014_6306_MOESM1_ESM.pdf]

<u>Additional File 1: Supplementary methods, tables and figures</u>

Section 1: Supplementary methods

a) Sequencing and phasing the *DEFA1A3* flanking region

A 4.1kb region flanking the *DEFA1A3* locus (GRCh37/hg19 chr8: 6876778-6880877) was sequenced in the 90 HapMap CEU1 individuals. The region was amplified as two overlapping fragments (3.2kb and 2.7kb) using the primers TGGGAGGCATAGGAGTTTCCA and CCCAGAAAACAGCATGGCATC or GCCTCCCCATGAAACTCAGAACC and TCTCACCAAGACCGTGCCTTCTG; these were designed to ensure amplification specifically from the *DEFA1A3* centromeric partial repeat. The region was amplified in a 20μl reaction, containing 1x One*Taq* buffer, at a One*Taq* DNA polymerase concentration of 0.25U/μl (NEB), 1 μM each primer and 20ng genomic DNA. The cycling conditions consisted of an initial denaturation at 94°C for 30 seconds, 30 cycles of 94°C for 30 seconds and 68°C for 5 minutes, followed by a final extension at 68°C for 5 minutes. For the vast majority of sequence variants observed, phasing could be inferred using segregation, as the region was sequenced in trios. However, there were some occasions where allele-specific PCR was required to determine the phase. 1μl of a 1:10 dilution of the amplified region was reamplified in a 10μl reaction containing 1μM each primer, 0.5 Units *Taq* DNA polymerase (NEB) and a standard buffer (see Methods section). The primers and cycling conditions are shown in table S4. These products were sequenced to allow the phase to be determined.

b) Using read depth to estimate *DEFA1A3* copy number

The 1000 Genomes project provides whole-genome sequence data for 1047 independent individuals [1]. Using read depth analysis, it has been possible to estimate the *DEFA1A3*

copy number of each sample. However, it is not clear how accurate this method of copy number estimation is, in comparison to the PCR-based methods used by Khan *et al.* [2], which have been used to accurately determine *DEFA1A3* copy number. For 84 of the 1000 Genomes samples, the *DEFA1A3* copy number had been estimated by Khan *et al*. [2]. These values were compared to each other, showing a high correlation between the two values (figure S1). For 95% of samples, read depth estimated the *DEFA1A3* copy number to be the same or one integer different to that determined by Khan *et al*.[2], suggesting that read depth provides an accurate measurement of copy number.

Section 2: Supplementary tables

Table S1: The D’ and r^2 values for the four SNPs flanking the *DEFA1A3* locus that tag *DEFA1A3* haplotype class. Values calculated based on diploid genotype calls for the HapMap CEU and HRC1-5 samples with European ancestry.

SNP1	SNP2	D'	r^2
rs7825750	rs62487514	1	0.06
rs7825750	rs4300027	1	0.40
rs7825750	rs7826487	1	0.06
rs62487514	rs4300027	1	0.15
rs62487514	rs7826487	1	0.02
rs4300027	rs7826487	1	0.14

Table S2: The number of HapMap CEU and HRC individuals with each combination of *DEFA1A3* haplotype class and frequency of a feature of the *DEFA1A3* copy number variable region (i.e. *DEFA1A3* copy number or *DEFA3,* Indel5 insertion or 7bp duplication frequency). These values were used for Chi Square and Cochran-Armitage tests comparing *DEFA1A3* haplotype class with features of the *DEFA1A3* locus. ++= homozygous positive, + - = heterozygous, - - = homozygous negative. Not all 599 individuals were included in each test, as some failed quality control analysis [2].

<table>
<tr><th>DEFA1A3 haplotype class</th><th colspan="6">DEFA1A3 Copy Number</th><th colspan="2">DEFA3 freq</th><th colspan="6">Indel5 insertion freq</th><th colspan="2">7bp duplication freq</th></tr>
<tr><td>Reference Sequence</td><td colspan="3">3-7</td><td colspan="3">8-16</td><td>0-1</td><td>2-7</td><td colspan="3">0-2</td><td colspan="3">3-6</td><td>0-3</td><td>4-11</td></tr>
<tr><td>+ +</td><td colspan="3">9</td><td colspan="3">61</td><td>18</td><td>47</td><td colspan="3">56</td><td colspan="3">13</td><td>53</td><td>17</td></tr>
<tr><td>+ -</td><td colspan="3">137</td><td colspan="3">133</td><td>126</td><td>126</td><td colspan="3">194</td><td colspan="3">72</td><td>203</td><td>66</td></tr>
<tr><td>- -</td><td colspan="3">199</td><td colspan="3">48</td><td>140</td><td>70</td><td colspan="3">128</td><td colspan="3">101</td><td>102</td><td>134</td></tr>
<tr><td>Class 1</td><td colspan="2">3-6</td><td colspan="2">7-8</td><td colspan="2">9-16</td><td>0-1</td><td>2-7</td><td colspan="2">0-1</td><td colspan="2">2-3</td><td colspan="2">4-6</td><td>0-3</td><td>4-11</td></tr>
<tr><td>+ + / + -</td><td colspan="2">85</td><td colspan="2">47</td><td colspan="2">7</td><td>99</td><td>27</td><td colspan="2">15</td><td colspan="2">101</td><td colspan="2">21</td><td>82</td><td>58</td></tr>
<tr><td>- -</td><td colspan="2">129</td><td colspan="2">209</td><td colspan="2">100</td><td>185</td><td>216</td><td colspan="2">184</td><td colspan="2">175</td><td colspan="2">68</td><td>276</td><td>159</td></tr>
<tr><td>Class 2</td><td colspan="3">3-7</td><td colspan="3">8-16</td><td>0-1</td><td>2-7</td><td colspan="3">0-2</td><td colspan="3">3-6</td><td>0-3</td><td>4-11</td></tr>
<tr><td>+ +</td><td colspan="3">46</td><td colspan="3">2</td><td>2</td><td>42</td><td colspan="3">45</td><td colspan="3">2</td><td>30</td><td>18</td></tr>
<tr><td>+ -</td><td colspan="3">19</td><td colspan="3">62</td><td>114</td><td>117</td><td colspan="3">185</td><td colspan="3">60</td><td>170</td><td>79</td></tr>
<tr><td>- -</td><td colspan="3">110</td><td colspan="3">167</td><td>168</td><td>84</td><td colspan="3">148</td><td colspan="3">124</td><td>158</td><td>120</td></tr>
<tr><td>Exchange 1</td><td colspan="3">3-7</td><td colspan="3">8-16</td><td>0-1</td><td>2-7</td><td colspan="3">0-2</td><td colspan="3">3-6</td><td>0-3</td><td>4-11</td></tr>
<tr><td>+ +</td><td colspan="3">3</td><td colspan="3">13</td><td>15</td><td>0</td><td colspan="3">0</td><td colspan="3">16</td><td>0</td><td>16</td></tr>
<tr><td>+ -</td><td colspan="3">59</td><td colspan="3">52</td><td>83</td><td>14</td><td colspan="3">22</td><td colspan="3">89</td><td>13</td><td>98</td></tr>
<tr><td>- -</td><td colspan="3">284</td><td colspan="3">166</td><td>186</td><td>229</td><td colspan="3">356</td><td colspan="3">81</td><td>345</td><td>103</td></tr>
<tr><td>Exchange 2</td><td colspan="2">3-6</td><td colspan="2">7-8</td><td colspan="2">9-16</td><td>0-1</td><td>2-7</td><td colspan="2">0-1</td><td colspan="2">2-3</td><td colspan="2">4-6</td><td>0-3</td><td>4-11</td></tr>
<tr><td>+ + / + -</td><td colspan="2">44</td><td colspan="2">43</td><td colspan="2">16</td><td>70</td><td>21</td><td colspan="2">62</td><td colspan="2">31</td><td colspan="2">4</td><td>77</td><td>26</td></tr>
<tr><td>- -</td><td colspan="2">170</td><td colspan="2">213</td><td colspan="2">91</td><td>214</td><td>222</td><td colspan="2">137</td><td colspan="2">245</td><td colspan="2">85</td><td>281</td><td>191</td></tr>
</table>

Table S3: The number of 1000 Genomes individuals with each combination of *DEFA1A3* haplotype class and *DEFA1A3* copy number. These values were used for Chi Square and Cochran-Armitage tests comparing *DEFA1A3* haplotype class with *DEFA1A3* copy number for the ASN, AFR, AMR and EUR samples. There were no tests comparing *DEFA1A3* copy number with Exchange 1 in the AFR and ASN samples, as this class is absent in the ASN samples and only two examples have been observed in the AFR samples. ++= homozygous positive, + - = heterozygous, - - = homozygous negative.

<table>
<tr><th>DEFA1A3 haplotype class</th><th colspan="6">Africa</th><th colspan="6">Asia</th><th colspan="6">America</th><th colspan="6">Europe</th></tr>
<tr><th>Reference Sequence</th><th colspan="3">3-7</th><th colspan="3">8-17</th><th colspan="3">3-7</th><th colspan="3">8-17</th><th colspan="3">3-7</th><th colspan="3">8-17</th><th colspan="3">3-7</th><th colspan="3">8-17</th></tr>
<tr><td>+ +</td><td colspan="3">1</td><td colspan="3">3</td><td colspan="3">12</td><td colspan="3">20</td><td colspan="3">6</td><td colspan="3">9</td><td colspan="3">6</td><td colspan="3">40</td></tr>
<tr><td>+ -</td><td colspan="3">34</td><td colspan="3">23</td><td colspan="3">60</td><td colspan="3">79</td><td colspan="3">41</td><td colspan="3">36</td><td colspan="3">53</td><td colspan="3">112</td></tr>
<tr><td>- -</td><td colspan="3">109</td><td colspan="3">58</td><td colspan="3">67</td><td colspan="3">39</td><td colspan="3">67</td><td colspan="3">16</td><td colspan="3">133</td><td colspan="3">21</td></tr>
<tr><th>Class 1</th><th colspan="2">3-6</th><th colspan="2">7-8</th><th colspan="2">9-17</th><th colspan="2">3-6</th><th colspan="2">7-8</th><th colspan="2">9-17</th><th colspan="2">3-6</th><th colspan="2">7-8</th><th colspan="2">9-17</th><th colspan="2">3-6</th><th colspan="2">7-8</th><th colspan="2">9-17</th></tr>
<tr><td>+ + / + -</td><td colspan="2">49</td><td colspan="2">61</td><td colspan="2">24</td><td colspan="2">2</td><td colspan="2">1</td><td colspan="2">53</td><td colspan="2">16</td><td colspan="2">20</td><td colspan="2">5</td><td colspan="2">33</td><td colspan="2">29</td><td colspan="2">17</td></tr>
<tr><td>- -</td><td colspan="2">43</td><td colspan="2">32</td><td colspan="2">19</td><td colspan="2">74</td><td colspan="2">93</td><td colspan="2">54</td><td colspan="2">59</td><td colspan="2">44</td><td colspan="2">31</td><td colspan="2">89</td><td colspan="2">109</td><td colspan="2">88</td></tr>
<tr><th>Class 2</th><th colspan="3">3-7</th><th colspan="3">8-17</th><th colspan="3">3-7</th><th colspan="3">8-17</th><th colspan="3">3-7</th><th colspan="3">8-17</th><th colspan="3">3-7</th><th colspan="3">8-17</th></tr>
<tr><td>+ +</td><td colspan="3">3</td><td colspan="3">5</td><td colspan="3">21</td><td colspan="3">1</td><td colspan="3">13</td><td colspan="3">1</td><td colspan="3">43</td><td colspan="3">1</td></tr>
<tr><td>+ -</td><td colspan="3">58</td><td colspan="3">27</td><td colspan="3">75</td><td colspan="3">51</td><td colspan="3">62</td><td colspan="3">13</td><td colspan="3">102</td><td colspan="3">56</td></tr>
<tr><td>- -</td><td colspan="3">83</td><td colspan="3">52</td><td colspan="3">43</td><td colspan="3">86</td><td colspan="3">39</td><td colspan="3">47</td><td colspan="3">47</td><td colspan="3">116</td></tr>
<tr><th>Exchange 1</th><td colspan="6" rowspan="4">N/A</td><td colspan="6" rowspan="4">N/A</td><th colspan="3">3-7</th><th colspan="3">8-17</th><th colspan="3">3-7</th><th colspan="3">8-17</th></tr>
<tr><td>+ +</td><td colspan="3">0</td><td colspan="3">3</td><td colspan="3">1</td><td colspan="3">4</td></tr>
<tr><td>+ -</td><td colspan="3">9</td><td colspan="3">13</td><td colspan="3">27</td><td colspan="3">38</td></tr>
<tr><td>- -</td><td colspan="3">105</td><td colspan="3">45</td><td colspan="3">164</td><td colspan="3">131</td></tr>
<tr><th>Exchange 2</th><th colspan="2">3-6</th><th colspan="2">7-8</th><th colspan="2">9-17</th><th colspan="2">3-6</th><th colspan="2">7-8</th><th colspan="2">9-17</th><th colspan="2">3-6</th><th colspan="2">7-8</th><th colspan="2">9-17</th><th colspan="2">3-6</th><th colspan="2">7-8</th><th colspan="2">9-17</th></tr>
<tr><td>+ + / + -</td><td colspan="2">52</td><td colspan="2">35</td><td colspan="2">26</td><td colspan="2">33</td><td colspan="2">48</td><td colspan="2">27</td><td colspan="2">28</td><td colspan="2">22</td><td colspan="2">9</td><td colspan="2">34</td><td colspan="2">27</td><td colspan="2">7</td></tr>
<tr><td>- -</td><td colspan="2">40</td><td colspan="2">58</td><td colspan="2">17</td><td colspan="2">43</td><td colspan="2">46</td><td colspan="2">80</td><td colspan="2">47</td><td colspan="2">42</td><td colspan="2">27</td><td colspan="2">88</td><td colspan="2">114</td><td colspan="2">98</td></tr>
</table>

Table S4: The primers and PCR conditions used for the allele-specific reamplification of regions flanking the *DEFA1A3* locus using the SNPs shown.

SNP	Primers	PCR Conditions		
rs62487509	GAGGGCTGCTGGAACAA	95°C	30 seconds	30x
	or GAGGGCTGCTGGAACAG	68°C	30 seconds	
	with AGCCCATTTGGATTGAAGCCT	70°C	1 minute	
rs4284061	CCCACTTGTCCATTCTGCA	95°C	30 seconds	26x
	or CCCACTTGTCCATTCTGCT	69.5°C	30 seconds	
	with Fseq (table S5)	70°C	2 minutes	
rs11137085	GTGAAATGGAGAGGTGTGGTC	95°C	30 seconds	25x
	or GTGAAATGGAGAGGTGTGGTG	69.5°C	30 seconds	
	with Rseq (table S5)	70°C	90 seconds	
rs4512398	CAGATCAGGCCAGCTCATGAGA	95°C	30 seconds	30x
	or AGATCAGGCCAGCTGATGAGG	68°C	30 seconds	
	with TGGGATCAGGCGCTAGTGAA	70°C	1 minute	

Table S5: The twelve primers used for the sequencing of the 4.1kb region flanking the *DEFA1A3* locus.

Primer Name	Sequence
Fseq	TGCAAKGCTCCAACTCTTCAG
Rseq	CCAGGTTTCTGCAGGACACACT
Rseq2	CCTCACTACCGTCCACCACAA
Rseq3	AGCAGGACCACRAGGCTTTT
RF4_2	GCCTCCCCATGAAACTCAGAACC
RR5	CACTGATTGTCTACACTGGCTGCAA
RR4	CAGGGACTTGGAGCTCCTACCTGT
RR3	TCTACAGGGGCACTCATTCCATTCA
RR2	TCCTCCTCCAAGCATGGTATCTGG
RR_2	GGACTGTGCGAAAAGACACCACA
RR	TCTCACCAAGACCGTGCCTTCTG
FE	CACCTGCAAGGATGGGCTAGAGA

Table S6: Primers, PCR cycling conditions and digest information for the RFLPs genotyping three SNPs tagging *DEFA1A3* haplotype classes. [a] The forward primer introduces a mismatch (underlined) creating a control *Rsa*I site, whilst the reverse primer introduces a mismatch (underlined) creating a context in which the SNP position is part of a variable *Rsa*I site. [b] the reverse primer introduces a mismatch (underlined) creating a context for the SNP to make a variable *Tsp*509I site.

SNP	Primers	Cycling Conditions	Product Size and Digest
rs7826487	CCTTCATTCCTTCCACCAGA<br>and<br>ACACCACCCCTGCTCTGAAC	95°C 1 minute<br>95°C 30 seconds<br>64.5°C 1 minute 35x<br>70°C 1 minute	1021bp<br>*Ssp*I digest<br>G= 251bp + 770bp<br>A= 251bp + 611bp + 159bp
rs7825750[a]	GGATTGCAGCAGGTTTATTGTAC<br>and<br>CATGTAGTCTGTTGGTCAAAATGGT	95°C 1 minute<br>95°C 30 seconds<br>56.5°C 30 seconds 33x<br>70°C 30 seconds<br>70°C 5 minutes	165bp<br>*Rsa*I digest<br>C= 21bp + 144bp<br>T= 21bp + 119bp + 25bp
rs62487514[b]	ATCTCTCTTTGGATGGTG<br>and<br>GGTTCATATACAACATCTTCTTAAT	95°C 1 minute<br>95°C 30 seconds<br>50°C 30 seconds 32x<br>70°C 1 minute<br>70°C 5 minutes	233bp<br>*Tsp*509I digest<br>C= 45bp + 188bp<br>A= 45bp + 162bp + 26bp

Table S7: Primers and PCR cycling conditions used for each of the four EHF-PCR systems. The assays were based on a design described by Tyson and Armour [3].

<table>
<tr><th>EHF-PCR System</th><th>Primers</th><th>PCR cycling conditions</th></tr>
<tr><td>Telomeric Gene</td><td>F1: TTGTGAAAACATGGCCCACCT
F2'R1: TTGCAGAATACCAGCGTGCATTAAAGCACCCATGTGCCTAGT
R2: CGGGAGAGAGGTTCCAGAGTT</td><td rowspan="3">98°C 30 seconds
98°C 10 seconds ⎤
71°C 30 seconds ⎬ 40x
72°C 1 minute ⎦
72°C 5 minutes</td></tr>
<tr><td>Centromeric Gene</td><td>F1: GCAGTGGGTGGGGAAATCAG
F2'R1: TTCTCTAGCCCATCCTTGCAGGCGGGAGAGAGGTTCCAGAGTTG
R2: TGGTGTTGGCTCAGCTGGAA</td></tr>
<tr><td>Telomeric Indel5</td><td>F1: TTGTGAAAACATGGCCCACCT
F2'R1: GCTCTCCCTCTTCCTGGACAGTGAAAGCACCCATGTGCCTAGT
R2: CCAGCTGGAGGGTCTCTGTTCT</td></tr>
<tr><td>Centromeric Indel5</td><td>F1: CACTGTCCAGGAAGAGGGAGAGC
F2'R1: TTCTCTAGCCCATCCTTGCAGGCCAGCTGGAGGGTCTCTGTTCT
R2: TGGTGTTGGCTCAGCTGGAA</td><td>98°C 30 seconds
98°C 10 seconds ⎤
71°C 30 seconds ⎬ 40x
72°C 15 seconds ⎦
72°C 5 minutes</td></tr>
</table>

Table S8: Primers and PCR cycling conditions for the allele-specific reamplification of EHF-PCR products, using the SNPs shown.

SNP	Primers	PCR cycling conditions
rs2978951<br>Telomeric Indel5<br>and Telomeric Gene	CGTTCGATCACATGAGTGGA or GTTCGATCACATGAGTGGG with GGTTCCAGAGTTGGGTCTCA (Telomeric Gene) or TCTGGGTGCTGCATGTAAAGC (Telomeric Indel5)	95°C 30 seconds<br>67°C 30 seconds<br>70°C 1 minute<br>35x
rs2738046<br>Telomeric Indel5<br>and Telomeric Gene	CTCATCTGCCCCCTTCCA or CTCATCTGCCCCCTTCCC with GGTTCCAGAGTTGGGTCTCA (Telomeric Gene) or TCTGGGTGCTGCATGTAAAGC (Telomeric Indel5)	95°C 30 seconds<br>68°C 30 seconds<br>70°C 1 minute<br>35x
rs4512398<br>Centromeric Indel5	AGGCCAGCTCATGAGA or AGGCCAGCTCATGAGG with GAAGAGGGAGAGCGGGTG	95°C 30 seconds<br>65°C 30 seconds<br>70°C 15 seconds<br>35x
rs4512398<br>Centromeric Gene	AGGCCAGCTCATGAGA or AGGCCAGCTCATGAGG with TGAAGCCCAAACTCCTGCTTG	95°C 30 seconds<br>65°C 30 seconds<br>70°C 1 minute<br>35x
rs17382102<br>Centromeric Indel5	TTAACCACAGAAATTTACAAACAT or TTAACCACAGAAATTTACAAACAC with GAAGAGGGAGAGCGGGTG	95°C 30 seconds<br>69°C 30 seconds<br>70°C 15 seconds<br>35x
rs17382102<br>Centromeric Gene	TTAACCACAGAAATTTACAAACAT or TTAACCACAGAAATTTACAAACAC with TGAAGCCCAAACTCCTGCTTG	95°C 30 seconds<br>69°C 30 seconds<br>70°C 1 minute<br>35x

Section 3: Supplementary figure

Figure S1: ***DEFA1A3* number estimates.** Comparison of *DEFA1A3* copy number estimates obtained by Khan *et al*. [2] and read depth analysis for 84 HapMap CEU samples. For the majority of samples (69%), the integer copy number calls agree.

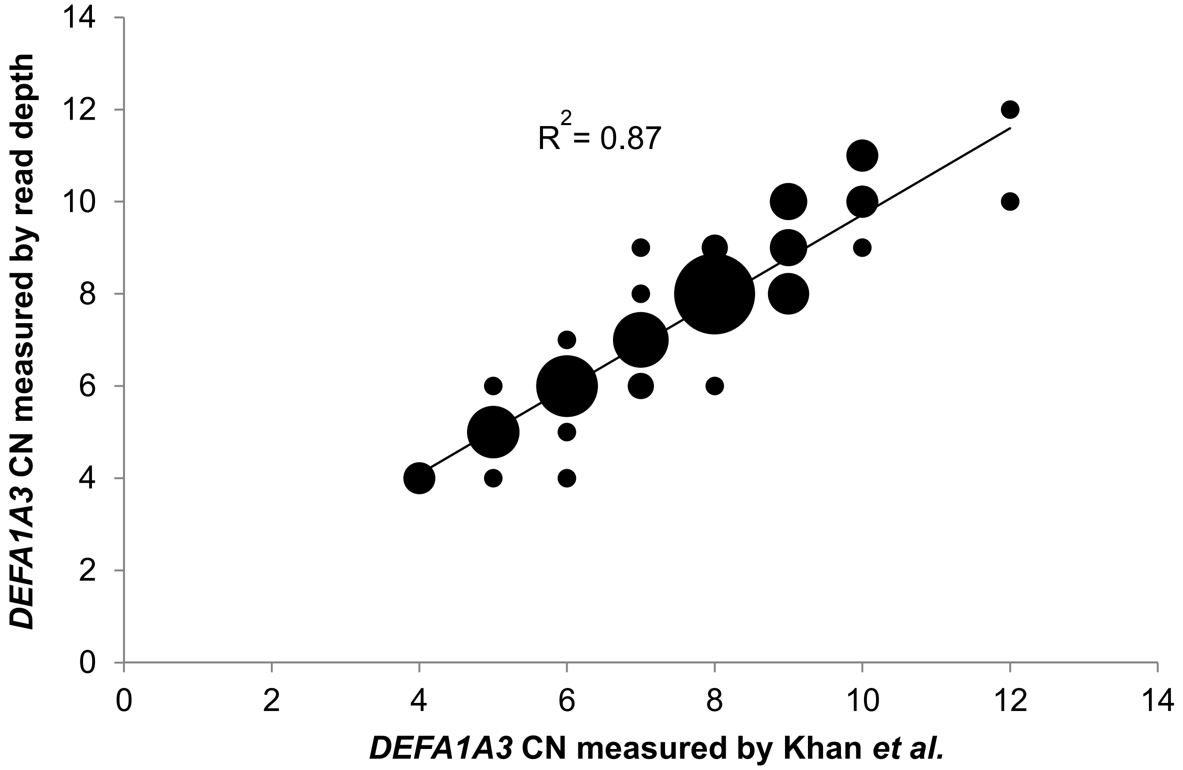

References

1. Altshuler DM, Durbin RM, Abecasis GR, Bentley DR, Chakravarti A, Clark AG, Donnelly P, Eichler EE, Flicek P, Gabriel SB *et al*: **An integrated map of genetic variation from 1,092 human genomes**. *Nature* 2012, **491**(7422):56-65.
2. Khan FF, Carpenter D, Mitchell L, Mansouri O, Black HA, Tyson J, Armour JA: **Accurate measurement of gene copy number for human alpha-defensin DEFA1A3**. *BMC Genomics* 2013, **14**:719.
3. Tyson J, Armour JA: **Determination of haplotypes at structurally complex regions using emulsion haplotype fusion PCR**. *BMC Genomics* 2012, **13**:693.
